# When is local excision appropriate for “early” rectal cancer?

**DOI:** 10.1007/s00595-013-0766-3

**Published:** 2013-11-21

**Authors:** Kotaro Maeda, Yoshikazu Koide, Hidetoshi Katsuno

**Affiliations:** Department of Surgery, Fujita Health University School of Medicine, 1-98 Kutsukake, Toyoake, Aichi 470-1192 Japan

**Keywords:** Early rectal cancer, T1 rectal cancer, T2 rectal cancer, Stage 1 cancer, Local excision

## Abstract

Local excision is increasingly performed for “early stage” rectal cancer in the US; however, local recurrence after local excision has become a controversial issue in Western countries. Local recurrence is considered to originate based on the type of tumor and procedure performed, and in surgical margin-positive cases. This review focuses on the inclusion criteria of “early” rectal cancers for local excision from the Western and Japanese points of view. “Early” rectal cancer is defined as T1 cancer in the rectum. Only the tumor grade and depth of invasion are the “high risk” factors which can be evaluated before treatment. T1 cancers with sm1 or submucosal invasion <1,000 μm are considered to be “low risk” tumors with less than 3.2 % nodal involvement, and are considered to be candidates for local excision as the sole curative surgery. Tumors with a poor tumor grade should be excluded from local excision. Digital examination, endoscopy or proctoscopy with biopsy, a barium enema study and endorectal ultrasonography are useful for identifying “low risk” or excluding “high risk” factors preoperatively for a comprehensive diagnosis. The selection of an initial local treatment modality is also considered to be important according to the analysis of the nodal involvement rate after initial local treatment and after radical surgery.

## Introduction

Local excision was performed in 5.9 % of the 13,434 patients with rectal cancer in the Swedish rectal cancer registry from 1995 to 2003, and the proportion of local excision in various procedures remained constant during that period [1]. Local excision was performed in 12.0 % of 2,131 patients with stage 1 (T1 and T2, N0, M0 [2]) rectal cancer in this registry from 1995 to 2001 [3]. On the other hand, the use of local excision increased in patients with stage 1 rectal cancer from 1989 to 2003 according to the National Cancer Database in the US [4]. Local excision was performed in a significantly higher proportion of patients with T1 lesions (37.9 %) than T2 lesions (12 %) during the period studied. The rate of local excision significantly increased, both for T1 lesions (26.6 % in 1989 vs. 43.7 % in 2003) and T2 lesions (5.8 % in 1989 vs. 16.8 % in 2003) [4].

Local excision is a procedure which can eliminate defecation, sexual and urinary dysfunction and the risk of a permanent stoma, with a shorter hospital stay and minimal mortality and morbidity because it avoids radical surgery [4–6]. However, due to the significant risk of local recurrence and a poorer prognosis after local excision compared with radical resection, its use and indications have recently become highly debated issues [4–11]. The key to potentially curative local treatment for rectal cancer is to select a suitable patient or tumor for local excision and to choose the most suitable local excision procedure. This review focuses on the inclusion criteria for “early” rectal cancers for local excision from the Western and Japanese points of view.

A literature review was undertaken using the MEDLINE database for the English literature and the Igaku Chuo Zasshi for the Japanese literature, and by cross-referencing previous publications. The appropriate publications were selected and cited for the review. The following key words were used for the searches: early rectal cancer, T1 rectal cancer, T2 rectal cancer, stage 1 cancer, local excision and local treatment.

## Definition of “early” rectal cancer and the rectum in Japan and Western countries

Rectal cancer has been defined as a cancerous lesion located within 12 cm of the anal verge by rigid proctoscopy in US [12, 13]. On the other hand, tumors with distal extension to <15 cm (as measured by rigid sigmoidoscopy) from the anal margin are classified as rectal, while more proximal tumors are classified as colonic in the ESMO guidelines [14]. The rectum is divided into two parts in Japan; the rectum above and below the peritoneal reflection, and the upper limit of the rectum above the peritoneal reflection is the lower end of the second sacral bone [15]. Therefore, the definition of the rectum differs according to the guidelines, rules and countries studied. However, the definition of the rectum itself is not considered to dramatically affect the outcomes of local excision for “early” rectal cancer, which is going to be discussed here, although it might be associated with the specific procedure adopted for local excision and the outcomes of more advanced rectal cancers.

T1 and T2 rectal cancers according to the tumor node metastasis classification (TMN) [2] have been reported as “early” [9, 16, 17] or “early stage” [18, 19] cancer in many Western publications. On the other hand, Tis and T1 cancers of the rectum have been considered as “early” cancer [15] in accordance with the Japanese classification of gastric cancer [20]. In the ESMO clinical practice guidelines [14], rectal cancer is divided into four groups: very early (some cT1), early (cT1–2, some cT3), more advanced (cT3, some cT4) and locally advanced (cT4). Neoplastic cells, when confined to the colorectal mucosa, are correctly defined as dysplasia or adenoma in the UK, and the misnomers “intramucosal carcinoma” and “carcinoma in situ” are used in the US and Japanese literature [7]. Tytherleigh et al. [7] defined early rectal cancer as invasive adenocarcinoma spreading into, but not beyond, the submucosa; that is, a T1 tumor in the TNM classification according to Morson’s definition [21]. This definition was used in several papers [22–24] and is adopted in this article as the proper definition of “early” rectal cancer. Stage 1 in the TNM classification includes T1 and T2 tumors with N0 and M0. It is currently impossible to correctly diagnose lymph node metastasis perioperatively when performing local excision [5, 7]. Therefore, T1 and T2 rectal cancers are both considered to be “early stage” rectal cancer in this article.

## Frequency of T1 and T2 tumors

Stage 1 (T1 and T2, N0, M0) rectal cancer comprises 20−34 % of all rectal cancers [3, 25–27]. Among the 35,179 patients with stage 1 cancer treated from 1989 to 2002 in the US, T1 lesions were identified in 43.8 % and T2 in 56.2 % of the patients [4]. Among 2,177 patients undergoing bowel resection for T1 and T2 lesions from 1995 to 1998 in Japan, T1 lesions were identified in 36.7 % of the cases [25]. The incidence of T1 lesions removed by endoscopic polypectomy in Japan rose from 3.8 % in 1978 to 10.3 % in 1997 [28]. Three to 8.6 % of all resected colorectal adenocarcinomas are reported to be T1 lesions in Western countries [16, 21, 29, 30], 3.6 % in Korea [31] and 12.1 % in Japan [25]. Tytherleigh and Mortensen et al. [7] stated that the incidence of early rectal cancer (T1 cancer) will likely rise following the start of the UK screening program. When considering the results of a national survey showing that colorectal cancers found at the time of screening are mostly early-stage cancers; with 45.3 % being cancer in situ, 20 % being T1 cancer and 11.1 % being T2 cancer [32], the number of “early” cancers is expected to increase not only in Japan, but also worldwide.

## Subclassification of T1 cancer

Haggitt’s subclassification [33] is most commonly used for polypoid T1 cancers or malignant rectal polyps (T1 cancer) in Western countries [13, 14]. It is based upon the extent of invasion of the stalk and is divided into five levels; 0: the absence of invasive carcinoma, 1: invasion into the head of the polyp, 2: invasion into the neck, 3: invasion into the stalk, 4: invasion into the base [33]. The subclassification of T1 cancers based upon the depth of invasion into the submucosal layer is also used for sessile-type tumors in both Japan [22, 34] and Western countries [14, 34]. In this subclassification, the submucosal layer is divided into three layers according to the depth of invasion; the 1: upper third, 2: middle third and 3: lower third [34]. The ESMO clinical practice guidelines [14] add that Haggitt’s levels 1–3 correspond to sm1, and level 4 may be sm1–3. Measurement of the depth of invasion into the submucosal layer is recommended by the General Rules for Clinical and Pathological Studies on Cancer of the Colon, Rectum and Anus in Japan [15], and additional bowel resection is recommended based on the distance of invasion into the submucosal layer after local treatment [25]. The term sm “scant” or “slight” is sometimes used as a term corresponding to sm1, and sm “massive” as sm3 (sometimes including sm2) in Japan. Very early cancer (some cT1) in the ESMO guidelines is considered to correspond to sm “scant” or “slight” T1 cancer (sm1) in Japan.

## “Risk factors” for T1 cancers

The risk factors and/or unfavorable criteria for T1 cancers can be defined as predictive factors for positive lymph nodes, tumor recurrence and decreased cancer-specific survival after local excision. Many clinical and pathological factors have been reported as risk factors. However, most of these factors can only be identified in the resected specimens after the resection of the tumor. Therefore, those factors could be useful as an indication for immediate salvage surgery or additional further treatments for local excision.

### Depth of wall invasion

The depth of wall invasion or the T stage is often reported to be closely associated with regional lymph node metastasis [6], and is one of the few factors which could be studied preoperatively. Haggitt’s level 4 invasion into the submucosa has been defined as a risk factor for lymph node metastasis [33]. Suzuki et al. [35] reported that the positive predictive value for nodal metastasis increases from 17 to 30 percent when the width (5 mm) of submucosal invasion is added to Haggitt’s level 4. The details of the association between the grade of invasion and regional lymph node metastasis will be discussed in the following sections.

### Tumor grade

The tumor grade has been demonstrated to be a significant indicator for lymph node metastasis and local recurrence [6], and is a factor that can be confirmed preoperatively. Poorly differentiated cancer [31, 36, 37], mucinous adenocarcinoma [36] and signet ring cell carcinoma [37] are reported to be risk factors for lymph node metastasis in T1 cancer. However, the proportion of tumors showing these histologies is low in all T1 tumors studied: 2.4 % [31], 6 % [36] and 7.3 % of all local excision cases [37]. Nascimbeni et al. [29] reported that poorly differentiated carcinoma was found to be the significant predictor of lymph node metastasis in a univariate analysis, but not in the multivariate model. Goldstein et al. [38] reported that the risk of lymph node metastasis was 0 % for Grade I tumors, 20 % for Grade II and 43 % for Grade III tumors. However, T2 tumors were included in the analysis performed for that study.

### Lymphatic and vascular invasion

Lymphovascular invasion has been reported to be a significant factor predicting lymph node metastasis in both univariate and multivariate analyses [29, 39–41], and lymphatic and blood vessel invasions were identified as independent factors predicting lymph node metastasis in several studies [17, 42, 43]. The presence of extramural vascular invasion [30] and vascular invasion [44] were also independent risk factors for nodal involvement in a multivariate analysis. Kaneko et al. stated that lymphatic involvement was a significant factor in a univariate analysis, but was not significant in a multivariate analysis [36]. Instead, the lymphatic vessel density at the site of deepest penetration was a significant independent factor predicting nodal involvement in the multivariate analysis, and they proposed that the identification of lymphatic vessels by podoplanin immunostaining provides an objective and accurate evaluation of lymphatic involvement. Brodsky reported that none of the T1 tumors without lymph vessel invasion or blood vessel invasion had lymph node metastasis [17].

### Tumor budding or sprouting

Tumor budding is characterized by the presence of tiny clusters of undifferentiated cells found ahead of the invasive front, and is also called as sprouting [7, 37, 41, 45, 46]. Tumor budding is another significant predictor of nodal involvement in several multivariate analyses [36, 37, 41, 44]. It might be reasonable to consider that the invasive front of the tumor might represent characteristics of the tumor or malignant potential. Kaneko et al. [36] showed that the lymphatic vessel density, as described in the former section, correlated with tumor budding and the degree of inflammation at the invasive front.

### Tumor location

Several reports have shown that early carcinoma located in the rectum has a higher rate of lymph node metastasis and/or local recurrence [33, 34, 47, 48]. Nascimbeni et al. [29] have indicated that the location in the rectum itself was not a significant risk factor when compared with other colonic segments in a study of 353 patients with sessile T1 lesions. However, when the rectum was divided into thirds, cancer in the lower one-third of the rectum was associated with a significantly higher risk of lymph node metastasis.

### Tumor size

Nascimbeni et al. [29] showed that the size of the carcinomas with nodal involvement was not significantly different from those without nodal involvement. In addition, carcinomas larger than 5 cm in diameter were found to have a higher rate of lymph node metastasis than carcinomas smaller than 5 cm in a univariate analysis, but not a multivariate analysis. Goldstein et al. [38] reported that the risk of nodal disease increased when the size was >3.5 cm, although their data included the specimens with T1 and superficial T2 tumors after abdominoperineal resection (APR). On the other hand, Graham et al. [49] reviewed 16 published series (94 % of tumors were T1 or T2 adenocarcinomas with no identified regional metastases) and identified that a tumor size greater than 3 cm was not a significant factor predicting local recurrence or a decreased survival. Several authors found that the size was not a significant predictive factor for nodal involvement and/or local recurrence [10, 17, 34, 50, 51].

### Configuration or morphology

Tumors that are sessile or flat type were found to be a risk factor for lymph node metastasis and local recurrence when compared with pedunculated tumors in T1 cancers [34]. Nonpolypoid growth (NPG) was associated with a significantly higher incidence of lymph node metastasis (29 %) than polypoid growth (PG) (7 %) when the pattern of tumor growth was classified as PG or NPG in T1 tumors [52]. Macroscopically, type IIc and IIa + IIc lesions were associated with a significantly higher incidence of lymph node metastasis (44 and 30 %) than type IIa and I lesions (4 and 8 %) [52]. Kitajima et al. [41] clarified that the rate of lymph node metastasis was 0 % in head invasion cases and stalk invasion cases with a submucosal (SM) depth <3,000 μm if the lymphatic invasion was negative for pedunculated T1 cancer, and it was also 0 % if the sm depth was <1,000 μm for nonpedunculated T1 tumors. Brodsky et al. [17] showed a trend toward decreased lymph node metastasis for sessile nonpedunculated tumors compared with nonpolypoid, exophytic or ulcerated lesions (*P* = 0.06) in pT1 and pT2 rectal cancers. The global survival and local recurrence rates were significantly better for patients with exophytic (polypoid and sessile) carcinomas than for those with non-exophytic (ulcerated and flat raised) lesions after a local procedure for rectal cancer, and the exophytic group included significantly more stage T1 and fewer T2 and T3 cancers in that report [53].

### Resection margin or circumferential positive margins

Positive surgical margins, unclear margins, and unknown or doubtful margins are not an uncommon condition after local excision for rectal cancer. The surgical margin-positive or unclear rate varies from 4.8 to 11.1 % in T1 tumors and from 9.8 to 22.5 % in T2 tumors, and it was 16.1 % in T1 and T2 cancers, 1.7 % in Tis and T1 tumors and 15.5 % in large, rectal villous adenomas based on several studies [4, 54–57]. Many authors showed that positive surgical margins were associated with a higher local recurrence rate (50–100 %) after local excision, mostly for T1 and T2 tumors [6, 11, 49, 58–61]. Morson showed that the 5-year local recurrence increased according to the grade of surgical excision; from 3 % with complete excision, to 14 % with doubtful excision and to 36 % with partial excision. They also noted that the global survival rate decreased with the grade of excision; which was 82 % with complete excision, 64 % with doubtful excision and 57 % with partial excision [61]. Graham et al. [49] reviewed a series of local excisions for rectal cancers and concluded that positive surgical margins were significantly associated with increased local recurrence and decreased survival. On the other hand, Paty et al. [11] could not demonstrate a correlation of positive margins with local control in a multivariate analysis, although their series included the cases with additional treatments after local excision. Positive surgical margins or circumferential margins are not considered to be a factor associated with the tumor characteristics or malignant potential, but is a factor closely related to the techniques or procedures adopted for local excision.

### Gender

Male gender was reported as one of the significant risk factors for lymph node metastasis of T1 cancers in the univariate and multivariate analyses [23]. On the other hand, female gender was a marginal risk factor (*P* = 0.059 and 0.076) for lymph node metastasis of T1 cancer in a univariate analysis, but not in a multivariate analysis [40, 41]. Koide reported a significantly higher rate of nodal involvement (*P* = 0.015) in female patients (21.9 %) than in male patients (5.3 %) in a univariate analysis of 108 patients with T1 colorectal cancer [62]. However, in other reports, gender was not confirmed as a significant predictor of nodal involvement in patients with T1 cancers in a multivariate analysis [31, 44].

### Combinations of risk factors

Ueno et al. [37] showed that an unfavorable tumor grade, definite vascular invasion, and tumor budding was the combination of qualitative factors that most effectively discriminated the risk for nodal involvement in T1 cancers, and that the nodal involvement rate was 0.7, 20.7 and 36.4 % in the no-risk, one-risk and multiple-risk factor groups. Koide showed that the sm invasion grade (sm1–2 and 3), lymph vessel and vascular vessel invasion and budding were significant high risk factors for nodal involvement, and that no cases with nodal involvement were observed if definitive lymph vessel, vascular vessel invasion and budding were not all confirmed, even in cases with sm 2 and 3 invasion [62].

Therefore, the possible “risk factors” for rectal cancer, which can be used preoperatively when deciding on the surgical option, local therapy or radical surgery, are the depth of invasion and tumor grade.

## Lymph node metastasis in T1 cancers according to the depth of invasion

The frequency of nodal involvement and/or metastasis in T1 cancer according to the grade of submucosal invasion is shown in Table 1 [25, 29, 34, 36, 39, 40, 42, 63]. The rate of nodal involvement and/or metastasis was 0–3.2 % in cases with a depth of invasion of sm1 or scant (corresponding to sm1), and 12–25 % in sm3 or massive invasion according to the subclassification of T1 cancer as described in the previous section [29, 34, 42, 63]. It was 8–11 % in cases with sm2 invasion [29, 34, 63].

**Table 1:** Literature reports of lymph node metastasis in T1 tumors according to the depth of invasion

References	*n*	Depth of invasion	Node positive (%)	*P* value
Kodaira et al. [63]	655	sm1	3.2	Not reported
	619	sm2	11.0	
	532	sm3	12.0	
Kikuchi et al. [34]	25	sm1	0	Not reported
	82	sm2	8.5	
	36	sm3	25.0	
Tanaka et al. [42, 52]	80	sm scant	2.5	<0.01
	97	sm massive	19.6	
Nascimbeni et al. [29]	70	sm1	3	0.001
	120	sm2	8	
	154	sm3	23	
Sakuragi et al. [39]	141	<2,000 μm	0.7	<0.001
	98	≥2,000 μm	15.5	
Yamamoto et al. [40]	166	sm1 + sm2	1.8	0.0004
	116	sm3	13.8	
Kaneko et al. [36]	65	<1,000 μm	1.5	0.0056
	203	≥1,000 μm	13.8	
JSCCR [25]	140	<1,000 μm	0	Not reported
	672	≥1,000 μm	12.8	

In the study by Yamamoto et al. [40], sm1 was defined as submucosal invasion up to 500 μm from the muscularis mucosa, sm2 as submucosal invasion between 500 and 1,000 μm and sm3 as invasion beyond 1,000 μm, and the nodal involvement rate of sm1 + sm2 was 1.8 %. When depth of submucosal invasion was measured, the rate of nodal involvement was 0–1.8 % if the depth of submucosal invasion was <1,000 μm [22, 34], and 12.8–13.8 % if it was ≥1,000 μm [25, 36, 40, 41]. Sakuragi et al. [39] showed that the nodal involvement rate was 0.7 % if the depth of submucosal invasion was <2,000 μm, and that it was 15.5 % if it was ≥2,000 μm. Yasuda et al. [44] demonstrated that there was no lymph node metastasis in T1 tumors invading <3,000 μm if no vascular invasion or tumor budding was confirmed. Yoshida et al. [64] reported in a study of 158 cases of T1 cancer that the depth of invasion of all the unidentified group cases was greater than 1,000 μm when sm cancer was classified into three groups based on the state of the muscularis mucosa (as the clearly identified group, identified group and unidentified group). The grade of sm massive invasion was described as one of the criteria indicating the need for additional bowel resection due to one of the risks of nodal involvement in the second edition of General Rules of Cancer of the Colon, Rectum and Anus published in 1980 [65], and sm massive invasion was defined in greater detail as deeper invasion beyond “sm slight invasion, for example, the invasion about 200 ~ 300 μm” in the fifth edition of General Rules published in 1994 [25, 66]. Finally, the depth of sm invasion as one of the risk factors was extended to >1,000 μm according to the results of accumulated case studies in the Japanese guidelines published in 2010 [25].

According to these data, we can conclude that T1 cancer with sm1 or submucosal invasion <1,000 μm is a “low risk” cancer in terms of the nodal involvement.

## Lymph node metastasis in T1 cancers according to the previous surgery

### The frequency of lymph node metastasis and lymph vessel invasion according to the initial treatment

The frequency of nodal involvement and lymph vessel invasion in patients undergoing radical surgery for T1 cancer according to the initial treatment method is shown in Table 2 [39, 67, 68]. The node-positive rate was 0–6.7 % in cases undergoing radical surgery after initial endoscopic or local resection, and it was 9.8–11.4 % in patients who had undergone initial radical surgery [39, 67, 68]. The frequency of lymph vessel invasion was 5.7–9.7 % in cases undergoing radical surgery after initial endoscopic resection, and it was 49–55.3 % after initial radical surgery [67, 68]. In these reports, the surgical margin-positive rate in cases with additional surgery after endoscopic resection was 42.9 (15/35 cases) and 51.2 (16/31 cases) %, respectively.

**Table 2:** The frequency of lymph node metastasis and lymph vessel invasion in patients undergoing radical surgery for T1 colorectal cancer according to the initial treatment

References	Initial treatment	*n*	Node positive (%)	Lymph vessel invasion
Inoue et al. [67, 106]	Endoscopically	15	6.7	5.7 (*n* = 35^a^)
	Surgery	35	11.4	55.3 (*n* = 38^a^)
Sawai et al. [68]	Endoscopically	31	0	9.7
	Surgery	51	9.8	49.0
Sakuragi et al. [39]	Local resection	147	4.5	NI
	Bowel resection	110	11.3	NI

*NI* not identified

^a^Based on the histological findings after the initial treatment

### Literature reports of lymph node metastasis in T1 cancer: a detailed analysis

The frequency of nodal involvement in patients undergoing radical surgery for T1 cancer according to the cases included is reported in Table 3 [25, 29, 31, 34, 36, 39–41, 69]. The node-positive rate widely varied from 6.3 to 20 % in the literature. Yamamoto et al. and Sakuragi et al. [65, 66] reported frequencies of 6.3 and 7.6 %, respectively. However, their reports included the cases after local resection. The frequency of nodal involvement after initial radical resection was 10.1–20 % [25, 29, 31, 34, 36, 41, 69].

**Table 3:** The literature reports of lymph node metastasis in T1 tumors

References	*n*	Inclusion criteria	Site	Node positive (%)
Kikuchi et al. [34]	108	Bowel resection	Colon and rectum	12.0
Nascimbeni et al. [29]	353	Colorectal resection	Colon and rectum	13.0
Sakuragi et al. [39]	278	Curative resection	Colon and rectum	7.6
Post-local resection included, *n* = 147				
Kitajima et al. [41]	865	Surgical resection	Colon and rectum	10.1
Yamamoto et al. [40]	301	Curative resection	Colon and rectum	6.3
Post-local resection included, *n*: unclear				
Kaneko et al. [36]	268	Surgical and endoscopic	Colon and rectum	10.8
Choi et al. [31]	168	Curative resection	Colon and rectum	14.3
Nash et al. [69]	145	Radical resection	Rectum	20.0
JSCCR [25]	800	Radical resection	Rectum	11.9

As shown in this section, the frequency of nodal involvement was lower if the patients undergoing additional radical surgery after endoscopic or local excision were included in the reports. This might be because additional radical surgery was performed when the surgical margin was positive, marginal or doubtful, even when the burn effect eliminated the residual cancer cells. Therefore, we can conclude that the selection of the initial treatment modality (local treatment or radical surgery), and the use of the proper local treatment method obtaining free surgical margins, is important. By doing this, we can eliminate unnecessary radical surgery.

## Lymph node metastasis and local recurrence after local excision in T2 cancers

### Literature reports of lymph node metastasis in T2 cancer

The literature reports of the nodal involvement rate in patients undergoing radical surgery for T2 cancer are listed in Table 4 [5, 17, 25, 30, 70–73]. The node-positive rate varies from 14.5 to 25.7 % in the literature. Chok et al. [72] discussed that the variations in the rate of lymph node metastasis reported in the literature for intramural tumors or early cancers are due to differences in the included specimens (polypectomy, local excision and radical resection), and the number of lymph nodes retrieved. The median number of lymph nodes examined was ten (interquartile range 6–14) and the node-positive rate was 14.5 % in their study. The frequency of nodal involvement in other reports was around 20–25 %, as shown in Table 4.

**Table 4:** The literature reports of lymph node metastasis in T2 tumors

References	*n*	Inclusion criteria	Site	Node positive (%)
Brodsky et al. [17]	128	Surgical resection	Rectum	21.8
Sitzler et al. [70]	96	Surgery	Rectum	19.6
Fang et al. [71]	152	Surgical resection	Colon and rectum	18.4
Baxter et al. [5]	Review	–	Rectum	17–23
Chok et al. [72]	193	Resection	Colon and rectum	14.5
Rasheed et al. [30]	247	Oncological resection	Rectum	19.0
Kajiwara et al. [73]	244	Curative resection	Colon and rectum	22.1
JSCCR [25]	1377	Radical resection	Rectum	25.7

In a study to identify T2 colorectal cancer with a low risk of nodal involvement, a poorly differentiated component, grade II + III, high-grade lymphovascular invasion and a positive myxoid cancer stroma were demonstrated to be independent risk factors for nodal involvement in a multivariate analysis, and the incidence of nodal involvement increased with the number of risk factors; with 0 risk factors associated with a 3.8 % incidence, 1: 12.6 %, 2: 23.9 % and three factors being associated with a 48.8 % incidence of nodal involvement [73]. However, almost all of these factors could only be identified after local excision, and therefore can only be used to indicate the need for additional surgery after local excision for T2 tumors.

### Local recurrence after local excision for T2 cancers

Kajiwara et al. [73] recently summarized the local recurrence rates for T2 cancers treated with local excision, and the local recurrence rates ranged from 19 to 47 % in cases without adjuvant therapy and from 5 to 26 % in cases treated with adjuvant therapy. Garcia-Aguilar et al. [8] noted that there were similarities between the incidence of local recurrence after the local excision of rectal cancer and the expected incidence of lymph node metastasis, and that suggests that tumor failure occurs in regional lymph nodes.

## Preoperative biopsy and final histological findings

The tumor grade is one of the significant factors that predicts “high risk” T1 cancer, and is one of the key factors used to decide on the management of early rectal cancer, as shown in the previous section [6, 31, 36, 37]. However, almost all of the previous reports have shown the results of “high risk” T1 cancers retrospectively from definitive final histological results. Petrelli et al. [74] reported that the biopsy specimens of rectal cancer are prone to sampling errors, and may not be adequate for the assessment of these histological parameters. Bretagnol et al. [6] commented that it is important to keep in mind that some differences exist in the grade of tumors between the initial biopsy and the excised specimen, and cited that there was only a 30 % accuracy when grading biopsies in Grade 1 (well-differentiated) tumors reported by Takahashi et al. [75].

Pigot et al. [57] reported that colonic villous adenoma is associated with a significant incidence of malignancy, with foci of invasive cancer that are already present in 10−20 % of patients at the time of diagnosis [76–79], especially in the rectal area [77]. In their 207 consecutive patients surgically treated for apparently benign villous rectal adenoma, nine tumors (4 %) were diagnosed as invasive carcinoma and 28 tumors (14 %) were diagnosed as in situ cancer based on the final histological evaluation [57]. Maeda et al. [56] reported that one “high risk” T1 tumor (2 %) and 19 in situ cancers (39.6 %) were confirmed based on the final histology among 48 tumors diagnosed as adenoma from the initial preoperative biopsy. Haboubi et al. [80] reported that the incidence of malignant polyps as a proportion of all adenomas removed varies between 2.6 and 9.7 %, with an average of 4.7 %. Leslie et al. [81] reviewed the colorectal adenomas removed by endoscopy, and foci of malignancy were found in 0.2−8.3 % of the cases [82–85].

On the other hand, in the villous (papillary) adenomas or tumors diagnosed as adenoma preoperatively, T1 or invasive cancers were confirmed in 35.4−51.8 % of the tumors at the final histological evaluation [86–88]. Borschitz et al. [89] reported that 79 patients (65.8 %) out of 120 patients with T1 cancer based on the final histological findings underwent surgery after the diagnosis of an adenoma. It is well known that the frequency of cancer inclusion is associated with the size of the villous tumors, but the depth of invasion of villous tumors is often limited in Tis or T1 [77, 88].

Therefore, even when a preoperative biopsy shows an adenoma, it is necessary to keep in mind that invasive or T1 cancer might be included in the tumors, especially in villous tumors, when deciding on the indications for local excision.

## Preoperative diagnosis of early rectal cancer

### Digital rectal examination

The guidelines proposed by the American Society of Colon and Rectum Surgeons Practice Parameters for the treatment of rectal carcinoma recommend that proctosigmoidoscopy should be performed in conjunction with a digital rectal examination to determine the distance of the lesion from the anal verge, its mobility and to assess its position in relation to the sphincter complex as part of a full physical examination [90]. The accuracy of the assessment of the depth of invasion by a digital rectal examination has been reported to vary from 58 to 88 % [6, 91–102]. However, most of these reports included more invasive (advanced) tumors.

The tumors within reach of digital examinations can be characterized as mobile, tethered or fixed [102]. Patients with large and fixed tumors can be immediately excluded from consideration for local excision [103] because these often show invasion into the deeper layer. Tethered tumors often show massive invasion into the submucosal layer or deeper [56].

### Endoscopy

Endoscopy has not been included as a tool for the preoperative staging of early rectal cancer in most of the English literature [5, 6, 94, 103], but is used as a tool of complete assessment of the colon to evaluate synchronous cancers and polyps [90]. On the other hand, endoscopy has been used as one of the important tools for identifying and/or treating early rectal cancers in Japan [22, 56]. Spraying of the abnormal mucosa with a soluble ink, such as indigo carmine dye and control of the air transformation during colonoscopy, as well as magnifying colonoscopy, have been used for further evaluating early rectal cancer [7, 22, 104]. Saito et al. [105] reported the accuracy of the endoscopic diagnosis concerning the grade of T1 invasion to be 74.7 % (over-diagnosis rate 16.3 %, under-diagnosis rate 9.0 %). They demonstrated that the significant endoscopic findings showing invasion beyond 1,000 μm into the submucosal layer were expansion, hardness, irregularity, an uneven surface, fold convergence, the retraction and hardness of the circle in protruded tumors, a protruded lesion in a depressed lesion, an irregularity in a depressed lesion, strong redness, no deformity after air inflation and easy contact bleeding in superficial tumors, in addition to the finding of protruded tumors [105].

Inoue et al. [106] evaluated the efficacy of using the endoscopic findings after submucosal injection of 20 % glucose for diagnosing the depth of invasion of colorectal cancer, where the depth of invasion of the lesion lifted after injection was defined as Tis or sm1 (slight submucosal invasion), and that of not lifted (“non-lifting sign”) was sm2 and more. The sensitivity, specificity and overall accuracy were 91.2, 100 and 92.2 %, respectively, by this method. Hurstone et al. [107] evaluated the relationship between the invasive type V pit pattern using high-magnification chromoscopic colonoscopy and the submucosal invasion depth for flat and depressed colorectal lesions. They concluded that the pit pattern is useful for the in vivo staging of the depth of submucosal invasion in flat and depressed colorectal lesions, and that it is as sensitive as conventional 7.5 MHz EUS. However, there was a tendency to overstage lesions, and hence, the technique is limited by its low overall specificity. Nishigami et al. [108] demonstrated the significance of the desmoplastic reaction, which is proven by biopsy specimens, in the diagnosis of submucosal invasion of carcinoma of the colon and rectum. The desmoplastic reaction was not observed at the surface of sm1 tumors, but was seen in 68 out of 70 sm2 tumors and 74 out of 75 sm3 tumors after defining the submucosal invasion up to 500 μm from the muscularis mucosa as sm1.

### Double-contrast barium enema studies

Barium enema studies have also not been included as a tool for the preoperative staging of early rectal cancer in the English literature [5, 6, 94, 103], but it is useful to define the location, circularity and extension of rectal tumors, as well as being a tool for the complete assessment of the colon to evaluate synchronous cancers and polyps [90]. Double-contrast barium enema studies might be useful to identify sm massive tumors by the findings of retraction, defects or deformity of the rectal wall [56].

### Endorectal ultrasonography (ERUS)

Kwok et al. [109] reported that the accuracy of ERUS was 87 % for the T stage and 74 % for lymph node involvement in a systemic review of 53 studies. Schaffzin et al. [99, 102, 110–112] reviewed the accuracy of ERUS, and the accuracy for the T stage varied from 62 to 93 % and that for nodal involvement from 61 to 88 %. Bipat et al. reported that the sensitivity for lymph node involvement was 67 % in their meta-analysis of ERUS [113]. However, many of these reports were based on studies of all T grades. Garcia-Aguilar et al. reported that the accuracy of ERUS for T1 tumors was 47 %, that for T2 tumors was 68 % and that for T3 tumors was 70 %, and concluded that the accuracy of ERUS was operator-dependent and varied by tumor stage, with transmural and benign tumors the most likely to be accurately staged [114]. Landmann et al. [115] reported that the overall accuracy of ERUS nodal staging for their study cohort was 70 %, with a 16 % false-positive rate and a 14 % false-negative rate, and stated the limitations of EURS in accurately staging nodal disease for early rectal lesions.

On the other hand, the accuracy of differentiating between T1 and more advanced cancer by ERUS has been reported to range from 81 to 94 % [113, 116–119]. Stark et al. [116] reported that the sensitivity of ERUS with regard to invasive cancer was 89 %, the specificity was 88 %, the positive predictive value was 76 %, the negative predictive value was 95 % and the accuracy was 88 %, and among pT0 and pT1 tumors, the corresponding figures were 80, 88, 62, 95 and 87 %. They additionally stated that over-staging was more common in patients who had undergone a previous excision and in tumors with peritumoral inflammation and desmoplastic reaction.

Concerning the diagnosis of grade of T1 cancer by ERUS, Akasu et al. reported that the sensitivity/specificity/overall accuracy rates for the detection of slight submucosal invasion, massive submucosal invasion and muscularis propria invasion were 99/74/96, 98/88/97 and 97/93/96 %, respectively, and those for the detection of positive nodes were 53, 77 and 72 % [120]. Santoro et al. [121] reported that the depth of invasion was correctly determined in 87.2 % of both pT1-slight and pT1-massive lesions using high-resolution three-dimensional ERUS, and concluded that this method is useful for assessing the depth of invasion in early rectal cancer and for selecting the therapeutic options.

### Computed tomography (CT) and magnetic resonance imaging (MRI)

CT and MRI of the chest and abdomen are useful tools for identifying metastasis to the lungs and the liver [7, 102, 103]. However, the primary limitation of CT is that it cannot define the layers of the rectal wall, and thus, cannot assess the depth of penetration of the lesion [102]. In many reports, the accuracy of CT in assessing the depth of invasion and/or nodal involvement was worse than that of ERUS [94, 102, 122, 123]. The accuracy of CT for predicting nodal involvement varies widely, from 22 to 73 % [124]. Several authors commented on the limitations of CT as a method of staging rectal cancer, especially in early rectal cancers [102, 103, 125].

The accuracy rates of the T grade and nodal involvement by MRI varies from 66 to 92 % and from 57 to 90 %, respectively [98, 102, 126–128]. ERUS and MRI appear to be equally good at assessing lymph node involvement [7, 113, 129]. In a review of 90 articles that reported on at least 20 patients with histological confirmation of the stage, Bipat et al. [113] reported that ERUS and MRI had similar sensitivities for T1 vs. T2 lesions, and the specificity of ERUS (86 %) was significantly higher than that for MRI (69 %), indicating that there was over-staging of T1 tumors by MRI. They reported similar sensitivity (67 vs. 66 %) and specificity (78 vs. 76 %) for the detection of nodal disease by ERUS and MRI. However, both examinations are highly operator-dependent [5, 113]. So far, there have been no reports differentiating slight invasive T1 tumors from massive invasive T1 tumors by CT or MRI.

Concerning preoperative tumor staging, Bretagnol et al. [6] concluded that ERUS was more accurate for local invasion, while the identification of lymph nodes remained a major point of concern.

## Current inclusion criteria for local excision in various guidelines

The practice parameters for the management of rectal cancer (revised) prepared by the standards practice task force of the American Society of Colon and Rectal Surgeons (published in 2013) recommended local excision as an appropriate treatment modality for carefully selected patients with T1 rectal cancers without high-risk features [90]. The criteria for local treatment include well to moderately differentiated T1 cancer, the absence of lymphovascular or perineural invasion and tumors less than 3 cm in diameter occupying less than one-third of the circumference of the bowel lumen [13, 90]. The NCCN clinical practice guidelines in oncology [13] showed that transanal excision can be indicated for the following rectal cancers: <30 % circumference of the bowel, <3 cm in size, clear margins (>3 mm), mobile, nonfixed, within 8 cm of the anal verge, T1 and T2 (using caution in T2 due to the high local recurrence rate), endoscopically removed polyps with cancer or indeterminate pathology, no lymphovascular or perineural invasion, well to moderately differentiated cancer and no evidence of lymphadenopathy on pretreatment imaging. In addition, these guidelines comment that when the tumor can be adequately identified in the rectum, transanal microsurgery may be indicated.

The ESMO clinical practice guidelines show that in the earliest, most favorable cases, chiefly the malignant polyps [Haggitt 1–3, T1 sm1 (-2?) N0], a local procedure, e.g., using the transanal endoscopic microsurgery (TEM) technique, is appropriate, and that the resection should be radical (R0), and that no sign of vessel invasion or poor differentiation should be present [14].

The Association of Coloproctology of Great Britain and Ireland (2012), “Federation Nationale des Centres de Lutte contre le Cancer” (FNCLC, initially published 1999) and “Association Francaise de Chirurgie” (AFC, revised in 2005) proposed similar guidelines for local surgery, indicating that local excision for a cure in rectal cancer should be restricted to T1 tumors with well or moderate differentiation and that are <3 cm in diameter [6].

An expert panel designated by the American College of Radiology showed that the optimal candidates for a local excision alone include small (<4 cm), low-lying T1 tumors without adverse pathological features [130].

On the other hand, the guidelines proposed by the Japanese Society for Cancer of the Colon and Rectum (JSCCR) describe very short inclusion criteria for local excision: preoperatively diagnosed Tis and slightly invasive T1 tumors located below the peritoneal reflection, adding that TEM can excise more proximal tumors than conventional local excision procedures [25]. Instead, a more detailed description is provided for the inclusion criteria for endoscopic resection: preoperative diagnosed Tis and slightly invasive T1 cancer, tumors less than 2 cm and tumors with an undetermined morphology. In addition, these guidelines comment that the tumor histology must be evaluated. Expanding features, erosion, ulceration, fold convergence, deformity and hardness of the tumors are listed as endoscopic parameters, thus indicating the presence of massive invasive T1 tumors in the guidelines [25, 131]. The guidelines also recommend referencing the findings of a barium enema study, endoscopic observation with the use of dye, high-magnification endoscopic observation and ERUS for the further diagnosis of the depth of invasion [25, 132–134].

## Goals of local excision

Local excision for rectal cancer has many merits, because it can eliminate defecation, sexual and urinary dysfunction, and can prevent the need for a permanent stoma. It is also associated with a shorter hospital stay, and minimal mortality and morbidity because it avoids radical surgery [5, 6, 9, 94, 103, 135–137]. However, the relatively high recurrence rate has become an issue that has hindered the further application of the local excision of rectal cancer [7, 9]. The local recurrence rate after local excision alone varies widely, especially according to the T grade; from 9.7 % (range 0–24 %) for T1 cancer, 25 (range 0–67) percent for T2 and 38 (range 0–100) percent for T3 cancer [136]. To assess the true merits of local excision, equivalent local control with radical surgery has to be accomplished after local excision for rectal cancer.

The previous reports of local recurrence after local excision and radical surgery for T1 tumors are listed in Table 5 [4, 9, 69, 138–146]. Although most of these studies were retrospective studies, the local recurrence rate after local excision for T1 cancer ranged from 0 to 24 %, and that of radical resection from 0 to 9.9 %. In most cases, patients who underwent local excision tended to be older and had tumors located closer to the anal verge than those treated by radical surgery. The overall recurrence rate after radical resection for T1 rectal cancer was 1.1 % in the Japanese registry [25]. The selection of the patients and tumors, selection of the surgical procedure and the surgical margin-positive rate are considered to be the main factors responsible for this wide range of local recurrence after local excision [147].

**Table 5:** Literature reports of local recurrence after local excision and radical surgery for T1 tumors

References	Local excision		Radical surgery	
	*n*	Local recurrence (%)	*n*	Local recurrence (%)
Winde et al. [146]	24	4.1	26	0
Balani et al. [145]	7	0	17	5.9
Mellgren et al. [9]	69	18	30	0
Lee et al. [144]	52	4.1	17	0
Nascimbeni et al. [143]	70	6.6	74	2.8
Bentrem et al. [142]	151	15	168	3
Endreseth et al. [141]	256	12	35	6
You et al. [7]	601	12.5	493	6.9
Ptok et al. [140]	120	6	359	2
De Graaf et al. [139]	80	24	75	0
Nash et al. [69]	137	13.2	145	2.7
Peng et al. [138]	58	11	66	2

On the other hand, the local recurrence rate after radical surgery was less than 4 % in nine out of the 12 reports listed in Table 5. Furthermore, the nodal involvement rate was 0–3.2 % when the depth of submucosal invasion was slight, sm1, scant or <1,000 μm as discussed in the section about lymph node metastasis, according to the depth of invasion. The main difference between local excision and radical surgery is the omission of lymph node dissection [148], and radical surgery can excise a wide range of the rectum without the risk of surgical margin-positive early rectal cancer. Therefore, the goal of local excision for T1 rectal cancer as a sole curative surgery is to obtain a local recurrence rate equivalent to that of radical surgery or a nodal involvement rate of sm1 by selecting the proper tumors and surgical procedure to obtain clear surgical margins.

## When is local excision indicated for “early” rectal cancer?

As mentioned in the section about lymph node metastasis and local recurrence in T2 cancer, there is currently a limitation for the preoperative selection of “low risk” T2 cancers for local excision, and the nodal involvement of T2 cancers has been reported to be around 20–25 % (REFS). Therefore, T2 cancer is not considered to be a suitable tumor for local excision as the sole treatment method. Furthermore, there are many reports that have shown the limitations or undetermined effects of additional adjuvant therapy and salvage surgery with local excision for T2 cancers and “high risk” T1 cancers [6, 7, 9, 69, 73, 94, 130, 136, 147, 149–151].

As shown in the section about lymph node metastasis in T1 cancer based on the previous surgery, it is important to select the proper treatment modality; particularly whether local treatment or radical surgery should be performed as the initial surgery. Detailed preoperative staging is important for this purpose. However, the selection criteria for “low risk” T1 tumors are still an issue being discussed.

Practically, the only “risk factors” that can be diagnosed before surgery are the tumor grade and depth of wall invasion, as shown in the section about the “risk factors” for T1 cancers. Concerning the tumor grade, differentiated tumors should be confirmed preoperatively for their likelihood of being cured by local excision. However, preoperatively diagnosed adenoma can be considered a candidate for local excision, as mentioned in the section about preoperative biopsies and the final histological findings. As was discussed in the section describing the goals of local excision, tumors with sm1 invasion or submucosal invasion <1,000 μm are considered to be good candidates for local excision as a sole curative surgery.

In this era when the diagnosis of nodal involvement is not secure, as described in the section about the preoperative diagnosis of early rectal cancer, the diagnostic tools for determining “low risk” tumors with sm1 or submucosal invasion <1,000 μm, or for excluding “high risk” tumors with sm 2–3 or submucosal invasion >1,000 μm, should be used to select suitable tumors. Bretagnol et al. [6] concluded that the selection of patients should begin with a careful rectal examination, and that ERUS is currently the best method for preoperatively staging early rectal tumors to detect the depth of invasion (T stage) and lymph node status. Maeda et al. [56] reported that the selection for local excision was based on the findings from digital examinations, double-contrast barium enema studies, proctoscopy or colonoscopy with biopsy, as well as endoscopic ultrasonography, and that the selection criteria included: tumors without poor differentiation indicated by biopsy and tumors without “massive invasion” into the submucosal layer or deeper. The following findings were considered to indicate massive invasion: not mobile from the muscle layer by digital examination if palpable; prominent ulceration or a “non-lifting” sign on proctoscopy or colonoscopy; a retraction, defect or deformity of the rectal wall during a barium enema study and tumor extension close to the muscularis propria or deeper during endoscopic ultrasonography. These were mostly described in the section about the preoperative diagnosis of early rectal cancer. Thereafter, when the findings are not consistent for all examinations with regard to the preoperative diagnosis of the depth of invasion, local excision should be selected first as a total biopsy [57, 61], and the histological results can be awaited for the final diagnosis of the depth of invasion. Otherwise, radical surgery should be performed as the initial surgery. By following this strategy, the 12 patients reported in the study by Maeda et al. initially underwent radical surgery during the period studied, and 11 patients had “high risk” pT1 tumors, while the remaining one patient had Tis cancer.

We can conclude that a diagnosis excluding “high risk” cancers can be performed when deciding on the selection of local excision as a sole curative surgery or whether total biopsy should be performed. When diagnosing these cancers, digital examinations, double-contrast barium enema studies, proctoscopy and/or colonoscopy with biopsy and endoscopic ultrasonography are considered to be useful tools.

Local excision is also performed for patients unfit for major surgery because of medical comorbidities or in those with low lesions who are adamantly seeking sphincter preservation [149] even if tumors have “high risk” factors or were more advanced. In this situation, local excision cannot be performed as a sole curative surgery.

It is needless to say that selecting the proper local treatment modality that can obtain free surgical margins, and a proper histological evaluation after excision, are important parts of performing local excision for early rectal cancer [89].
